# Efficacy of a Smartphone-Based Virtual Companion to Treat Insomniac Complaints in the General Population: Sleep Diary Monitoring Versus an Internet Autonomous Intervention

**DOI:** 10.3390/jcm11154387

**Published:** 2022-07-28

**Authors:** Pierre Philip, Lucile Dupuy, Patricia Sagaspe, Etienne de Sevin, Marc Auriacombe, Jacques Taillard, Jean-Arthur Micoulaud-Franchi, Charles M. Morin

**Affiliations:** 1 SANPSY, UMR 6033, University of Bordeaux, F-33000 Bordeaux, France; patricia.sagaspe@chu-bordeaux.fr (P.S.); etienne.de-sevin@u-bordeaux.fr (E.d.S.); marc.auriacombe@u-bordeaux.fr (M.A.); jack.taillard@gmail.com (J.T.); jean-arthur.micoulaud-franchi@chu-bordeaux.fr (J.-A.M.-F.); 2 CNRS, SANPSY, UMR 6033, F-33000 Bordeaux, France; 3 Service de Médecine Universitaire du Sommeil, CHU de Bordeaux, Place Amélie Raba-Léon, F-33076 Bordeaux, France; 4 Bordeaux Population Health Research Centre, University of Bordeaux, Inserm, UMR U1219, F-33000 Bordeaux, France; lucile.dupuy@u-bordeaux.fr; 5 École de Psychologie, Université Laval, Québec, QC G1V 0A6, Canada; cmorin@psy.ulaval.ca; 6 Centre D’étude des Troubles du Sommeil, Centre de Recherche CERVO, Institut Universitaire en Santé Mentale de Québec, Québec, QC G1A 0A6, Canada

**Keywords:** insomnia, behavioral intervention, virtual agents, general population

## Abstract

(1) Background: Insomnia is the most prevalent sleep disorder worldwide and cognitive behavioral therapy is the front-line treatment. Digital health technologies have a role to play in screening and delivering interventions remotely and without the need for human intervention. The KANOPEE app, which provides a screening and behavioral intervention for insomnia symptoms through an interaction with a virtual agent, showed encouraging results in previous studies during and after the COVID-19 lockdown, but has not yet been evaluated in a controlled study. This study aims at comparing the benefits of KANOPEE, a smartphone application dealing with insomnia complaints, with another application proposing an electronic sleep diary named “My Sleep Diary”. The acceptance and potential benefits of these digital solutions are tested in real-life settings (i.e., without soliciting human medical resources) and in the general population. (2) Methods: Subjects were included if they downloaded one of the apps between December 2020 and October 2021, and were of legal age. Both apps were available on downloading platforms in France. Primary outcome was Insomnia Severity Index (ISI), and secondary outcomes were total sleep time (TST), sleep efficiency (SE) and wake time after sleep onset (WASO). (3) Results: A total of 535 users completed the intervention with KANOPEE and 489 users completed My Sleep Diary, both for 17 days. KANOPEE users improved their ISI score significantly more than sleep diary users (interaction Time*Group: F(2,2002) = 17.3, *p* < 0.001). Similar results were found for nocturnal sleep parameters (TST) (KANOPEE users gained 48 min of sleep after intervention, while My Sleep Diary users gained only 16 min of sleep), and particularly in the population with moderate to severe initial sleep complaints (F(4,1980) = 8.9, *p* < 0.001). Other sleep markers (SE and WASO) were significantly improved in the KANOPEE users compared to the sleep diary ones (*p* < 0.001). (4) Conclusions: KANOPEE provides significantly greater benefits than an electronic sleep diary regarding reduction of insomnia complaints and estimated nocturnal sleep characteristics in a self-selected sample of the general population. Population with the most severe initial ISI score (≥15) benefited the most from the KANOPEE App compared to filling up a simple sleep diary.

## 1. Introduction

Insomnia is the most prevalent sleep disorder. It is present in about 10 to 20% of the general population [1,2], and is frequently associated with mental conditions such as mood or anxiety disorders [3]. The ongoing COVID-19 crisis has increased the prevalence of mental disorders [4,5] and insomnia [6,7], confirming how sleep and mental disorders are closely related.

Most patients suffering from insomniac complaints receive hypnotic drugs, but medications are not recommended on a long-term basis because of their lack of long-term efficacy and potentially severe side-effects. The recommended first-line treatment for insomnia disorder in adults is cognitive behavioral therapies (CBT-I) [1]. These usually rely on multicomponent interventions including sleep hygiene education (e.g., avoiding caffeine and alcohol before bedtime), stimulus control and time in bed reduction (i.e., instructions aiming at strengthening the association between bed and sleep). In addition, cognitive interventions aimed at correcting negative thoughts and beliefs about sleep reinforce adherence to behavioral recommendations [1,8].

Unfortunately, due to the discrepancy between the huge number of patients needing treatment and the lack of trained healthcare practitioners, many patients do not have access to CBT-I. To combat this shortage, which has been accentuated by the COVID-19 crisis, autonomous digital solutions need to be promoted. These solutions, called digital CBT-I (d-CBT-I), have shown their efficacy in delivering CBT-I [9,10], reducing insomnia complaints and improving sleep efficiency [11,12,13,14,15]. More recently, d-CBT-I have also shown their benefit in reducing anxiety symptoms and improving psychological well-being [9,12]. 

Acceptance of d-CBT by users is also a key issue for promoting digital health solutions in the general population. Interaction with digital solutions can be improved by using embodied conversational agents, which have demonstrated a high level of acceptance in patients reporting mental disorders [16]. In 2020, our team launched a free app (KANOPEE) that is available on Google Play Store and Apple Store. Thanks to repeated interaction with a virtual companion, the app helps screen insomnia complaints, provides a follow-up tool (sleep diary) and gives users personalized recommendations regarding sleep hygiene, stimulus control, the optimization of sleep schedules and physical activity [17]. 

To date, KANOPEE has been downloaded more than 23,000 times, thus creating a large cohort followed by our research team. KANOPEE has shown a very good level of acceptance by users in the general population in a real-life setting [18]. A recently published open single-arm study of 108 KANOPEE users showed a decrease in insomnia complaints and an improvement in sleep duration and sleep efficiency [19]. However, the main limitation of that study was the lack of a controlled condition, precluding any definitive conclusions about whether sleep improvement is directly attributable to using KANOPEE.

To overcome this limitation, we designed a self-selected (non-randomized) controlled study comparing KANOPEE users to users of a non-interventional app, My Sleep Diary, an electronic sleep diary developed by our team and freely available on downloading platforms in France.

The objective of this study, conducted in a real-life naturalistic setting and offered to the general population, was to measure the efficacy of KANOPEE, a smartphone application proposing repeated interactions with a virtual agent to reduce sleep complaints over 17 days compared to another application giving access to an electronic sleep diary only. Based on the literature and previous studies, we hypothesized that both apps could reduce insomnia complaints and improve sleep, but that KANOPEE would have a greater impact on insomnia and sleep quality measures.

## 2. Materials and Methods

### 2.1. Design

The study design was a two-group non-randomized controlled trial with self-selection sampling. The first group of subjects downloaded and used KANOPEE, which provided interactions with a virtual companion to obtain personalized recommendations for improving sleep over 17 days. A second group of subjects downloaded My Sleep Diary to obtain access to an electronic sleep diary and a repeated follow-up on insomnia complaints, which was considered as the control condition (no intervention). 

Both apps were developed by our research lab, are downloadable for free on Google Play Store and Apple Store and are available only in France. A specific authorization from Google and Apple was obtained to put our non-profit apps online during the COVID-19 crisis. The study was approved by the ethical committee of University of Bordeaux and was registered at the US National Institute of Health (ClinicalTrials.gov) #NCT05074901. 

Methods, results and discussion are described using the CONSORT 2010 reporting guidelines.

### 2.2. Participants

Targeted communication promoting the usage of both apps to help with monitoring and regulating sleep was made on social media (Instagram, TikTok, Facebook, Twitter), national and regional newspapers and TV, and university and hospital mailing lists, in order to increase the download rate and reach the most diverse populations. The release of both apps was in line with government communication about the prevalence of sleep and mental complaints and as support to increase awareness of the importance of sleep during the COVID-19 crisis. 

From December 2020 to October 2021, subjects were included based on the following criteria: (1) having downloaded KANOPEE or My Sleep Diary; (2) having completed the first insomnia questionnaire between December 2020 and October 2021; and (3) being of legal age (aged 18 or older).

Criteria for not participating were: (1) not owning a smartphone; (2) not being located in France; (3) having completed the first insomnia questionnaire outside the inclusion period; or (4) being younger than age 18.

Recruitment was performed on Google Play Store and Apple Store by downloading one of the two apps. 

Baseline characteristics of users were collected at the registration step after acceptance of informed consent obtained directly on the apps, in accordance with General Data Protection Regulation (GDPR) and the Commission Nationale de l’informatique et des libertés (CNIL) requirements.

Both apps collected the users’ information about age (in years), gender (male/female), educational level (middle school/high school/less than 5 years of university/more than 5 years of university), initial insomnia complaints (Insomnia Severity Index (ISI), see later outcome measure) and depressive and anxiety symptoms (Patient Health Questionnaire-4 (PHQ-4)) [20].

All data were collected anonymously and stored on secure servers at University of Bordeaux. 

### 2.3. Interventions

Users in the intervention condition (KANOPEE) were invited to interact with Louise, a virtual companion providing screening, follow-up and personalized recommendations to decrease insomnia complaints.

#### 2.3.1. Screening Interview

**Figure 1:**
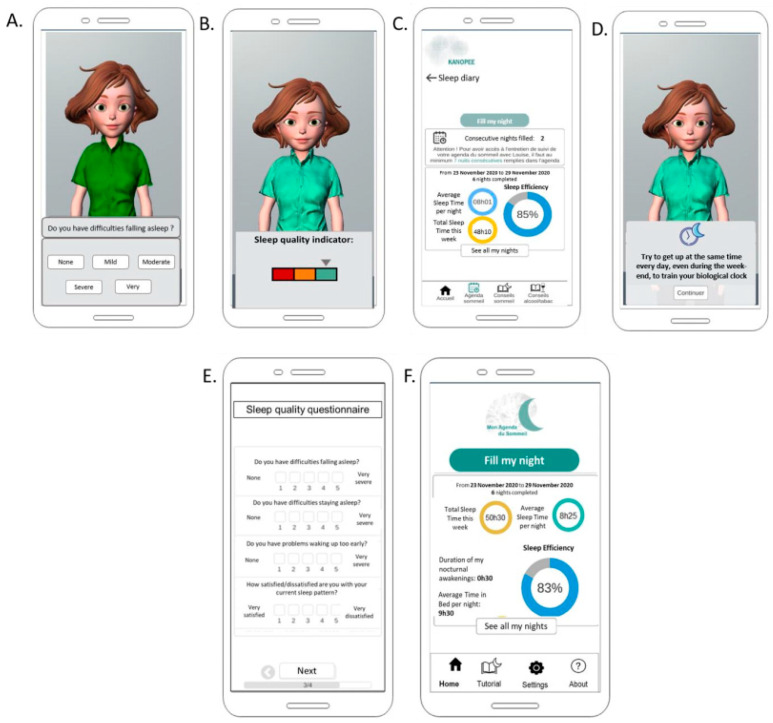
Examples of interfaces of KANOPEE (**A**–**D**) and My Sleep Diary (**E**,**F**) apps. (**A**) Screenshot of Louise questioning Insomnia Severity Index. (**B**) Screenshot of visual feedback given after screening interview with Louise. (**C**) Sleep diary and visual feedback on sleep patterns in KANOPEE. (**D**) Screenshot of personalized sleep recommendation given by Louise during follow-up interview. (**E**) Insomnia Severity Index administered in My Sleep Diary. (**F**) Visual feedback on sleep patterns in My Sleep Diary.

Screening consisted of administration of the Insomnia Severity Index (ISI) [21,22] (Figure 1A), then visual feedback (green/orange/red visual scale) (Figure 1B), and the offer of completing a sleep diary for one week and then having another interview at that time. After this screening interview, the sleep diary tab appeared on the bottom of the screen and users could fill it every day and obtain visual feedback on their sleep (Figure 1C).

#### 2.3.2. Follow-Up Interview

One week later, KANOPEE users were offered another interview with the virtual companion, who provided a summary of sleep diary data, administered the ISI for the second time, and then gave personalized recommendations based on ISI answers and on sleep diary data. The recommendations were simple sentences (e.g., “In the morning, expose yourself to sunlight or to another source of bright light (luminotherapy, screen) to improve the functioning of your biological clock”), associated with a picture (Figure 1D). Users were encouraged to follow these recommendations on sleep hygiene recommendation, stimulus control, time in bed restriction, relaxation and physical activity for 10 days and continue to use the sleep diary, and they were informed that they would have a third interview 10 days later. After this follow-up interview, a new tab “personalized recommendations” appeared at the bottom of the screen so that users could refer to them at any time.

#### 2.3.3. Final Interview

Ten days later, KANOPEE users had a last interview with Louise, who asked which recommendations users were able to follow, administered the ISI, and then, depending on the ISI score, gave the user the opportunity to continue using KANOPEE autonomously (if ISI ≤ 21) or be referred to a sleep specialist (if ISI > 21).

### 2.4. Control Condition

Users of My Sleep Diary had access to the exact same sleep diary proposed in KANOPEE (Figure 1F). Additionally, once a week, users could complete the ISI in a textual interface (Figure 1E). The differences compared to KANOPEE were the absence of a virtual companion administering the questionnaire, the absence of personalized recommendations, and no referral to a specialist if complaints persisted after two weeks of use. 

The potential bias induced by non-randomization was minimized as follows:

Both applications had the same “tags” on the download platforms (i.e., Apple Store and Google Play Store), so when a user searched for an app with the words “sleep” or “diary” or “insomnia”, they found both apps to download;

The inclusion period was the same for both apps in order to reduce any seasonal effects or those attributable to the COVID-19 pandemic;

If user characteristics at baseline differed from one group to another, further analyses were used to control the effect of those characteristics (age, gender, symptom severity, etc.) in the outcomes of interest.

To increase compliance, a reminder was sent every day from both apps to remind users to fill in their sleep diary, and every week to complete the ISI. Users did not receive any financial incentive for participation.

### 2.5. Outcome Measures

The primary outcome measure was the Insomnia Severity Index, a seven-item self-report questionnaire that provides a global measure of perceived insomnia severity (range from 0 to 28: 0–7 (no clinical insomnia), 8–14 (sub-threshold insomnia), 15–21 (insomnia of moderate severity), and 22–28 (severe insomnia)). The ISI has been validated and has proven sensitive to changes in insomnia severity with treatment [22]. In the intervention group (KANOPEE users), the ISI was administered by the virtual companion. In the control group (My Sleep Diary users), it was in a textual format. The secondary outcome measures included changes in nocturnal sleep indicators derived from the sleep diaries completed by the subjects: sleep onset latency (SOL), number of awakenings (NWAK), wake after initial sleep onset (WASO), terminal wakefulness (TWAK), total time spent in bed (TIB), total sleep time (TST), and sleep efficiency (SE). These sleep indicators have proven their validity [23,24]. The sleep diary was the same in both apps. Reminders sent every day in the event of non-completion reduced the amount of missing data. 

### 2.6. Statistical Analyses

#### 2.6.1. Sample Size

Sample size and power analysis were calculated based on our preliminary results [19] in which ISI scores differed between the first measure and the measure after one week of sleep diary intervention (15.60 ± 5.01 vs. 13.58 ± 5.21), representing an estimated Effect Size = 0.4. 

For an ANOVA with independent groups (sleep diary versus KANOPEE), a total sample size of 684 participants after the three-week follow-up is needed to obtain a sufficient statistical power (1-β err prob) = 0.80, an effect size = 0.10 (conservative hypothesis) and an α err prob = 0.05 (GPower). Considering the same rate of completion (10%) and frequency of downloads (700 downloads per month) as in our previous study, the inclusion period had to last at least 10 months to obtain the minimum total sample size of 684 users.

#### 2.6.2. Descriptive 

Quantitative variables were expressed as mean ± standard deviation (SD) and qualitative variables were expressed as percentage.

#### 2.6.3. Comparative Analyses

Univariate analyses with *t*-test comparisons for independent groups for continuous variables or Chi2 test for categorical variables were used to compare socio-demographic and clinical characteristics of participants between My Sleep Diary and KANOPEE groups. Four-way analyses of variance (ANOVAs) with repeated factor “Time” (“screening”: corresponding to scores at baseline, “after Step 1”: corresponding to scores after one week of app use; and “after Step 2”: corresponding to scores after 17 days of app use), and between subject-factors “Group” (sleep diary versus KANOPEE), “Severity of baseline insomnia complaints” (0–7, 8–14 and 15–28) and “Age” (Less than 50 years old, More than 50 years old) were used to analyze ISI and nocturnal sleep characteristics. Age categories were based on the median age of the study population. 

Chi2 tests were performed to compare the proportion of users in each severity category of insomnia complaints (none, mild, moderate to severe) by condition (sleep diary and KANOPEE) between first and last timepoint (screening versus after Step 2), and to compare the distribution of users’ severity of insomnia complaints between sleep diary versus KANOPEE at each timepoint (screening, after Step 1 and after Step 2). 

The alpha risk threshold was set at *p* = 0.05. Statistica^®^ (StatSoft Inc. 2010, Statistica for Windows, Maisons-Alfort, France, Version 9.1) was used.

## 3. Results

### 3.1. Participant Flow

**Figure 2:**
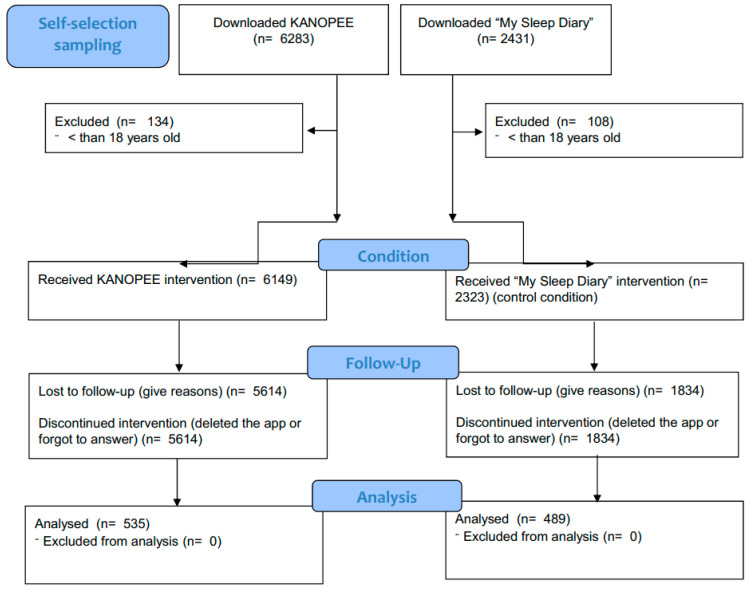
Participant flow.

The flowchart of participants can be found in Figure 2. During the inclusion period (i.e., December 2020 to October 2021), 8714 users downloaded the two apps (i.e., KANOPEE and My Sleep Diary), but 242 users were excluded for not meeting the inclusion criteria (age < 18 years old). Among the remaining 8470, 5614 KANOPEE users and 1834 My Sleep Diary users discontinued the intervention before the end (either forgot to answer or deleted the app) and were therefore excluded. Eventually, 535 KANOPEE users and 489 My Sleep Diary users were included in this study.

### 3.2. Characteristics of Participants

Table 1 shows baseline demographic and clinical characteristics of participants in the KANOPEE and sleep diary groups. They were predominantly female (two-thirds of the sample) with moderate to high educational level in both groups. KANOPEE users were significantly older (*p* < 0.001), scored slightly higher on insomnia complaints (ISI score, KANOPEE: 15.2 ± 4.5 vs. My Sleep Diary: 14.2 ± 5.2, *p* < 0.01) and expressed similar levels of depression and anxiety on the PHQ-4 questionnaire. There were more people without professional activity in the KANOPEE group than in the sleep diary group.

### 3.3. Insomnia Complaints

**Figure 3:**
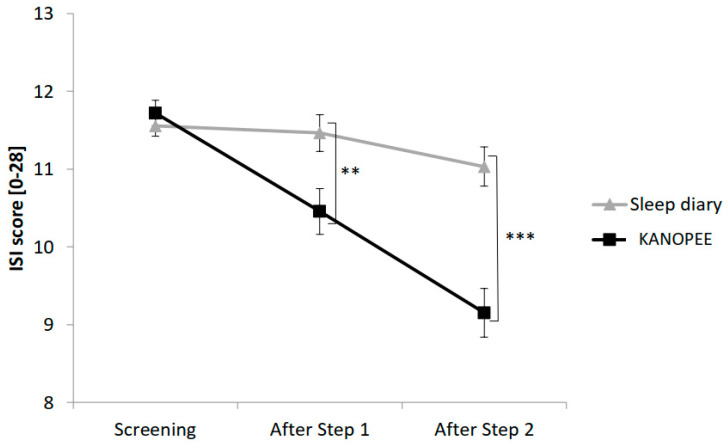
Insomnia severity index according to sleep diary versus KANOPEE over time. Note: Significance: **: *p* < 0.01; ***: *p* < 0.001.

The evolution of subjective insomnia complaints between the two groups at baseline, after Step 1 and Step 2 is presented in Figure 3. 

The main effect “Group” yielded significance for ISI score (F(1,1001) = 10.5, *p* < 0.001) with significantly lower ISI scores in the KANOPEE group than in the sleep diary group. A significant interaction between the “Group” factor and the “Time” factor (F(2,2002) = 17.3, *p* < 0.001) created a more marked reduction in ISI score over time in the KANOPEE group than in the sleep diary group (Figure 3).

**Figure 4:**
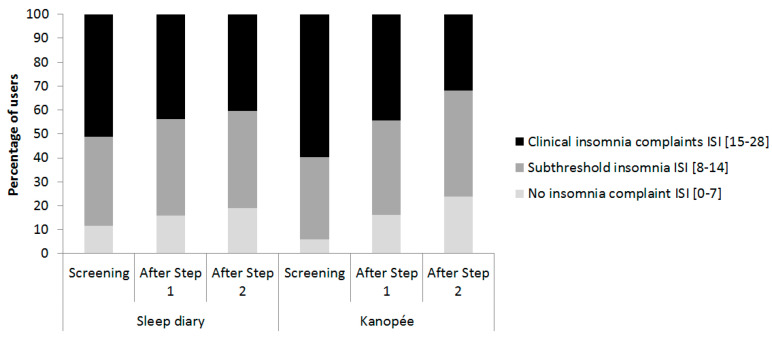
Distribution of sleep diary versus KANOPEE users depending on severity of their insomnia complaints over time.

Regarding the severity of baseline insomnia complaints, we obtained a significant interaction with the factor “Time” (F(4,2002) = 24.6, *p* < 0.001), indicating a greater decrease in ISI score over time for the most severe users, whatever the group. The distribution of KANOPEE and sleep diary users depending on the severity of their insomnia complaints over time is shown in Figure 4. The proportion of users with severe insomnia complaints decreased in both groups between the first and last timepoint (sleep diary: Chi2 = 179.2, ddl = 4, *p* < 0.001 and KANOPEE: Chi2 = 130.8, ddl = 4, *p* < 0.001). At screening, a higher proportion of users with severe insomnia complaints was observed in the KANOPEE group than in the sleep diary group (Chi2 = 13.3, ddl = 2, *p* < 0.01). There was no difference in distribution at the intermediate timepoint between the groups (Chi2 = 0.1, ddl = 2, NS). On the other hand, the trend was reversed after Step 2 with a greater proportion of less severe patients in the KANOPEE group than in the sleep diary group (Chi2 = 8.7, ddl = 2, *p* < 0.05). Consequently, a shift towards the lower classes in terms of severity of insomnia complaints seemed to occur over time in the KANOPEE group.

### 3.4. Nocturnal Sleep Indicators

Table 2 presents nocturnal sleep indicators of sleep diary and KANOPEE users over time.

Regarding sleep onset latency (SOL) and wake after sleep onset (WASO), a decrease was observed from screening to the last timepoint in both groups (respectively, F(2,1980) = 3.5, *p* < 0.05 and F(2,1980) = 6.6, *p* < 0.001). The interaction “Group”, “Time”, “Severity of baseline insomnia complaints” yielded significance (respectively, F(4,1980) = 5.9, *p* < 0.001 and F(4,1980) = 8.4, *p* < 0.001) showing that reduced SOL and WASO over time were observed mostly in KANOPEE users with severe initial insomnia complaints.

Time in bed (TIB) and terminal wakefulness (TWAK) were decreased from screening to the next two timepoints in both groups (respectively, F(2,1980) = 88.6, *p* < 0.001 and F(2,1980) = 6.5, *p* < 0.001). No difference was observed in TIB or TWAK reduction between the two groups. The severity of insomnia complaints did not influence the reduction in TIB or TWAK.

Regarding total sleep time (TST) and sleep efficiency (SE), scores increased in both groups from screening to the last timepoint (respectively, F(2,1980) = 10.4, *p* < 0.001 and F(2,2022) = 4.9, *p* < 0.001). The interaction “Group”, “Time”, “Severity of baseline insomnia complaints” yielded significance (respectively, F(4,1980) = 8.9, *p* < 0.001 and F(4,2022) = 9.3, *p* < 0.001), showing that increased TST and SE over time was observed mostly in KANOPEE users with severe initial insomnia complaints.

## 4. Discussion

The digital self-guided sleep health intervention provided by KANOPEE produced a significant and clinically meaningful reduction in insomnia symptom severity. This demonstrates that, in line with public health campaigns, the regular use of smartphone apps downloaded without a previous medical consultation can improve sleep complaints. This finding pleads for the idea of promoting autonomous digital interventions prior to members of the general public with sleep complaints consulting their general practitioner or specialized medical centers, especially during a period of tension marked by the COVID-19 crisis and the increasing emergence of sleep complaints in modern societies.

Furthermore, the KANOPEE intervention had a greater effect on sleep complaints than simply recommending subjects to monitor their sleep schedules by using a sleep diary. Both applications globally improved ISI scores and reduced TIB, but KANOPEE had a more significant benefit on sleep efficiency and increased TST. This effect can probably be explained by the personalized recommendations proposed in KANOPEE, targeting stimulus control and time in bed restriction, and consequently, reducing SOL and WASO over time. Interestingly, patients with severe insomnia at baseline (≥15 on ISI) improved significantly over time in the KANOPEE group, whereas severe patients in the sleep diary group did not change over time. The finding that an autonomous digital intervention can improve insomnia in severely affected patients, if they follow the recommendations for two weeks, is very encouraging for implementation in a large population of patients.

Several studies have already demonstrated the efficacy of digital CBT-I [9,11,12,13,14,15], but they were conducted as formal clinical trials with the rationale of participating in a research study. On the other hand, the KANOPEE project adopts an ecological public health perspective by providing help to the general population in a period when consultations with physicians are difficult to obtain. Its ecological design and its findings constitute a sound basis for using validated applications like KANOPEE and My Sleep Diary in members of the general population exposed to psychosocial stress.

By comparing the effects of using KANOPEE or a sleep diary, we obtained comparable self-monitoring of sleep quality and regular feedback, thus controlling for a placebo effect and reinforcing the validity of our results. A future development would be to conduct a controlled study with text-based interaction to highlight the advantages of interacting with virtual agents. While a randomized controlled study with two arms could have also provided interesting insights into the efficacy of both applications, it would suppose a selection process requiring human interactions, which is exactly what we want to avoid in this specific study. Our aim was to investigate the real-world perception of both apps (with no physician intervention) to understand how future campaigns could be undertaken to increase the download rate. The added value of using KANOPEE compared to a simple sleep diary would prove useful in public health campaigns.

An important limitation is that we did not conduct specific interviews to question KANOPEE users about which recommendations best improved their sleep because we wanted to leave the participants free to focus on their acceptance of the application during the 17 days of the program. Future research should investigate how interventions could be tailored to patients’ phenotypes and level of acceptance. Research in dedicated sleep centers would be more appropriate to explore this issue. Our results demonstrate a good efficacy on a 17-day period. It is worth considering further studies to demonstrate the efficacy of our apps on longer periods. It also could be interesting to demonstrate the efficacy of KANOPEE app on objective measures collected by actimetry or polysomnography.

The validity of interventions based on autonomous virtual agents at the population level is still a burning issue. However, our results in a sample of over 1000 subjects underline the potential for using these new autonomous digital solutions in medicine and the benefits that they represent for healthcare systems.

## Figures and Tables

**Table 1:** Socio-demographic and clinical characteristics of participants.

	Sleep Diary (*n* = 489)	KANOPEE (*n* = 535)	*p*-Value
Age, y. (M ± SD)	47.0 ± 13.6	51.2 ± 13.4	<0.001
Female (%)	69.3	64.1	NS
Educational level (%)			NS
Middle school	12.9	10.7	
High school	12.7	15	
Less than 5 y. of university	56.2	60.7	
More than 5 y. of university	18.2	13.6	
Professional status (%)			<0.001
Farmers	3.1	2.8	
Artisans	5.3	3.6	
Senior executive	38.2	36.8	
Middle management	13.1	9.7	
Employees	18.8	17.2	
Working class	2.2	0.7	
Retired	11	19.8	
Non-worker	8.2	5	
Student	0	4.3	
ISI initial score (M ± SD)	14.2 ± 5.2	15.2 ± 4.5	<0.01
ISI group (%)			<0.01
ISI (0–7)	11.5	5.8	
ISI (8–14)	37.2	34.6	
ISI (15–28)	51.3	59.6	
PHQ-4 initial score (M ± SD)			
Total score	5.5 ± 2.8	5.1 ± 3.3	NS

Notes. Y. = Years; M ± SD = Mean ± Standard Deviation; ISI = Insomnia Severity Index; PHQ-4 = Patient Health Questionnaire.

**Table 2:** Nocturnal sleep characteristics of participants on sleep diary and KANOPEE over time.

	Sleep Diary (*n* = 489)			KANOPEE (*n* = 535)		
	Screening	After Step 1	After Step 2	Screening	After Step 1	After Step 2
TIB (hh:mm) (M ± SD)	08:25 ± 01:21	07:51 ± 01:03	07:55 ± 01:02	08:37 ± 01:39	07:43 ± 01:04	07:47 ± 00:58
TST (hh:mm) (M ± SD)	06:41 ± 01:40	06:48 ± 01:04	06:57 ± 00:59	05:48 ± 02:23	06:15 ± 01:18	06:36 ± 01:05
SE (%) (M ± SD)	79 ± 17	81 ± 11	83 ± 10	68 ± 26	74 ± 14	74 ± 14
SOL (hh:mm) (M ± SD)	00:34 ± 00:47	00:30 ± 00:28	00:29 ± 00:29	00:45 ± 01:04	00:37 ± 00:29	00:31 ± 00:27
WASO (hh:mm) (M ± SD)	00:32 ± 00:43	00:31 ± 00:33	00:28 ± 00:28	01:06 ± 01:38	00:49 ± 00:41	00:39 ± 00:34
NWAK (number of events) (M ± SD)	1.4 ± 1.4	1.3 ± 0.9	1.2 ± 0.9	1.7 ± 1.3	1.5 ± 0.9	1.3 ± 0.8
TWAK (hh:mm) (M ± SD)	00:36 ± 00:48	00:34 ± 00:31	00:31 ± 00:27	00:57 ± 01:11	00:50 ± 00:43	00:40 ± 00:34

Notes. TIB = Time in bed; TST = Total sleep time; SE = Sleep efficiency; SOL = Sleep onset latency; WASO = Wake time after sleep onset; NWAK = Number of night-time awakenings; TWAK = Terminal wakefulness, Hh:mm = Hours minutes; M ± SD = Mean ± Standard Deviation.

## Data Availability

Not applicable.
